# Pharmaceutical cost control in primary care: opinion and contributions by healthcare professionals

**DOI:** 10.1186/1472-6963-9-209

**Published:** 2009-11-18

**Authors:** Alexandra Prados-Torres, Amaia Calderón-Larrañaga, Antoni Sicras-Mainar, Sebastià March-Llull, Bárbara Oliván-Blázquez

**Affiliations:** 1 Instituto Aragonés de Ciencias de la Salud, Avda Gómez Laguna 25, Pl 3a, 50009 Zaragoza, España; 2 Badalona Servicios Asistenciales SA, C/Gayetà Soler, 6-8 Entresuelo, 08911 Badalona, Barcelona, España; 3 Unidad de Investigación, Gerencia de Atención Primaria de Mallorca, Instituto Balear de la Salud, C/Reina Esclaramunda 9, 07003 Palma de Mallorca, Islas Baleares, España

## Abstract

**Background:**

Strategies adopted by health administrations and directed towards drug cost control in primary care (PC) can, according to earlier studies, generate tension between health administrators and healthcare professionals. This study collects and analyzes the opinions of general practitioners (GPs) regarding current cost control measures as well as their proposals for improving the effectiveness of these measures.

**Methods:**

A qualitative exploratory study was carried out using 11 focus groups composed of GPs from the Spanish regions of Aragon, Catalonia and the Balearic Islands. A semi-structured guide was applied in obtaining the GPs' opinions. The transcripts of the dialogues were analyzed by two investigators who independently considered categorical and thematic content. The results were supervised by other members of the team, with overall responsibility assigned to the team leader.

**Results:**

GPs are conscious of their public responsibility with respect to pharmaceutical cost, but highlight the need to spread responsibility for cost control among the different actors of the health system. They insist on implementing measures to improve the quality of prescriptions, avoiding mere quantitative evaluations of prescription costs. They also suggest moving towards the self-management of the pharmaceutical budget by each health centre itself, as a means to design personalized incentives to improve their outcomes. These proposals need to be considered by the health administration in order to pre-empt the feelings of injustice, impotence, frustration and lack of motivation that currently exist among GPs as a result of the implemented measures.

**Conclusion:**

Future investigations should be oriented toward strategies that involve GPs in the planning and management of drug cost control mechanisms. The proposals in this study may be considered by the health administration as a means to move toward the rational use of drugs while avoiding concerns about injustice and feelings of impotence on the part of the GPs, which can lead to lack of interest in and disaffection with the current measures.

## Background

The economic impact of drug prescription is one of the principal concerns of health administrations. This is common to most European countries, where drug expenditures as a proportion of the Gross Domestic Product (GDP) and of total health expenditure has increased during the last 30 years, and is expected to continue rising [1]. In Spain, as an example of a National Health System offering universal health coverage, prescription drug costs rose to almost 10 million euros in 2008, which represents a 6.2% increase from the costs in 2007 [2]. This increase is associated not only with an increase in the prevalence of chronic illnesses [3,4] but also with the manufacture of and demand for new and more efficacious drugs, which are more expensive [5,6].

In the primary care (PC) setting, drug prescriptions represent the most important capital expenditure, accounting for half of the total costs of family health services [7] and 10% of a national health system's expenditure [8]. There is considerable variability between healthcare professionals and health centres (HCs) with regard to the profile of prescription drugs [9,10]. Moreover, the literature indicates that there are ways to improve the efficiency of drug prescriptions in PC without damaging either the patient or the quality of the prescriptions [11] and this was corroborated by most doctors in a qualitative study carried out within the UK National Health Service [12].

To date, the principal strategies adopted by health administrations have been based on measures that are essentially economic, such as the regulation of drug prices and commercial margins, the review of public financing for specialty drugs, and the implementation of financial incentives to limit costs--e.g. drug budgets or performance based payments--[1,13,14]. The actual health care environment, which is based on the medical freedom of physicians and the emphasis of healthcare professionals on the effectiveness of treatment to the detriment of efficiency [15], does not promote the spontaneous development of certain activities such as reducing healthcare resource use or modifying clinical practice [16]. Incentive schemes may serve to respond to priority objectives of the health care system, such as the rational use of drugs [17,18].

In Spain, where the health administration is becoming increasingly decentralized, the implemented prescribing incentive schemes vary depending on the regional health service. They range from direct monetary incentives --bonuses/sanctions-- for compliance with budgetary constraints to greater access to training activities for healthcare professionals who meet the budgetary targets set by the health administration [11].

These measures can generate conflict between the perspective of health administrators and the clinical vision of general practitioners (GPs) [19], who often lack appropriate training on health management tools and the rational use of drugs [20], and who perceive these managerial interventions as a "top down" approach that can lead to a loss of clinical freedom and a deterioration of their relationship with patients [21]. Cost containment incentives can outshine the objective of promoting the rational use of medications and can influence GPs to prescribe drugs that are less effective and safe than others [22,23].

The present study collects and analyzes the opinions of PC physicians regarding current measures of drug cost control, as well as their proposals for improving the effectiveness of these measures. Although this study has a particular significance around the PC structure in Spain, the pressures on GPs to curb budgetary demands are not unique to Spain, but exist across a range of healthcare systems [14].

Qualitative methods are used for this purpose, since it enables deeper insight into behaviour and attitudes, thus being particularly valuable in the evaluation of health management strategies [24-26].

## Methods

A qualitative exploratory study was designed using focus groups in order to obtain information [27]. Eleven focus groups composed of GPs from the regions of Aragon, Catalonia and the Balearic Islands met between March and July 2007. All participants belonged to HCs that had been selected for a previous study related to the classification of health problems and the identification of health care demand patterns in PC. The participants were invited to participate by the coordinators of the HCs. Emphasis was placed on the voluntary nature of participation and on the independence of the study with respect to the health administration agencies. A total of 82 GPs and 11 HCs were enrolled in the study. The main characteristics of the participants are summarized in Table 1. Ethical approval for this study was granted by the Ethics Committee of Aragon (CEICA).

**Table 1:** Characteristics of the focus group participants

Focus group	Region	Participants; N	% female	Age range	Work experience*
**G1**	Aragon	7	71%	43-62	25.4 (6.9)

**G2**	Aragon	5	40%	40-50	22.0 (8.0)

**G3**	Aragon	8	38%	43-58	25.0 (5.9)

**G4**	Aragon	9	78%	28-56	13.6 (10.6)

**G5**	Balearic Islands	14	57%	30-61	17.7 (10.4)

**G6**	Balearic Islands	7	43%	35-53	21.6 (6.5)

**G7**	Balearic Islands	8	63%	39-62	23.6 (6.4)

**G8**	Catalonia	9	78%	28-47	6.8 (3.6)

**G9**	Catalonia	6	50%	28-55	15.5 (10.8)

**G10**	Catalonia	7	57%	28-50	6.7 (4.2)

**G11**	Catalonia	2	100%	34-43	13 (6.0)

*Mean and SD of years worked in primary care; HC = health centre.

The focus group sessions, which took place in the HCs, were supervised by two researchers from the team, one acting as moderator and the other as observer. The duration of the sessions ranged from 45 to 90 minutes. In all cases, dialogues were recorded with the permission of the participants and on the understanding that all information was confidential. Data for analysis were obtained from 10 of the 11 transcripts, one being excluded because it included an insufficient number of participants (only two persons).

The sessions followed a semi-structured guide based on two major issues: 1) current strategies of drug resource allocation and pharmaceutical cost control and 2) patient classification systems and their implementation in PC. As a result, opinions of secondary relevance were obtained from the interviews. This study analyzed this information in a structured manner, based on two main information-bearing blocks: 1) GPs' perceptions of current measures of drug cost control, with a special emphasis on incentives meant to result in pharmaceutical cost savings, and 2) proposals for improving this control.

Two investigators independently analyzed the data for categorical and thematic content, in accordance with the defined dimensions. The elements that stood out in each of the transcripts were identified and coded. The text data were divided into units and subsequently grouped in categories according to analogue criteria (similarities between different units) that tend to delimit the possible connections between them. Atlas.ti 5.0 software was used as a tool for ordering and coding the data. Data saturation was considered to have been reached when the information began to be redundant. The results were supervised by different researchers from the team, taking into account the context of interpretation by the team leader.

## Results

The results of this qualitative study are presented as a function of the two thematic blocks mentioned earlier: perceptions of GPs regarding current drug cost control mechanisms and their proposals aimed at improving the effectiveness of these measures.

### Perceptions of the pharmaceutical cost control mechanisms

GPs highlight their awareness of their *public responsibility *with respect to pharmaceutical costs.

"I believe there is an increasing awareness from the moment you start talking about public resources, public money, and the magnitude of the public budget. Let's say this is a new professional attitude" (G5)

"We are very used to being evaluated, more specifically here, and this is no longer something out of the ordinary, [...], there are many factors that go to show it is a good thing, [...], because we have an important public responsibility with respect to expenditure" (G5)

"I want to know what I spend the money on [...], resources are limited and if we use them for one thing, we take them away from another, I mean... I know my management capacity is very small but I believe we can all collaborate" (G1)

However, they also express feelings of frustration, impotence and injustice towards the current drug cost control measures implemented by health administrators. The *feelings of frustration and impotence *result from the fact that the tools at their disposal are insufficient to control some factors, including pressure exerted by patients, the pharmaceutical industry, and their own colleagues, as well as prescriptions written by specialists.

"We are under considerable pressure as a result of the advertising campaigns, the consumer society, and our own work-place, plus the pressure from the pharmaceutical laboratories. Often, things are beyond our control" (G7)

The *feelings of injustice *result from the fact that the greatest part of the cost control responsibility falls on the PC setting; instead, they feel other health care settings should share this responsibility more equitably.

"When I do not have 100% overall responsibility for this prescription, I am not to be penalized. If they penalize me, they should penalize the cardiologist or the gastroenterologist..." (G1)

This situation creates a lack of *motivation *and dissatisfaction with the current measures. The GPs feel that they end up merely dispensing prescriptions and referring patients to specialist care.

"I am here so that you send me to the dermatologist ... to have a blood test done ... to get a prescription. And I answer: madam, they would not have a doctor here, they would have a machine instead" (G3)

"They should be the ones dispensing the prescriptions; I am tired of being the secretary of specialist care ." (G2)

They affirm that they find the motivation to work on pharmacy cost control-related aspects within their own work teams, in which the role of the coordinator is a key element.

" [...] I have been in several health centres [...]. In some of them, they are much more insistent either because the coordinator pays more attention or because you face up more indicators. And there are others where the topic is never brought up [...]. It depends a lot on the centre and on the power of stimulation of the coordinator and the team itself" (G6)

At the same time, GPs believe that the current control mechanisms do not affect their *prescription habits*. They proceed according to their own criteria and are conscious of their principal commitment to the health and safety of their patients. This attitude is strengthened by their close relationship and frequent contact with their patients, as well as by their awareness of the value of follow-up.

"What we do is common sense. They tell me I have to give this or the other not to be penalized... I find this unacceptable. Drugs are our working tools. We are physicians and if I believe that it is the best that I can give, I shall give it" (G4)

"Of course, it is very different for these people (specialists) who do not get involved [...], who will hopefully see the patient once every 6 months, but us, who see them every day, we take the patients very much into account ..." (G6)

The topic of *financial incentives *gave rise to confrontational opinions in the focus group sessions. While some physicians said they consider it a source of encouragement, others contend that, given that their obligation is to properly carry out their job with a fair salary as the reward, it is not ethical for the "savings" to the health system to be directly connected to the latter in the form of a bonus salary.

"If a part of our income is in the form of economic incentives, there will be less discouraged physicians. I bet they suddenly regain the interest towards pharmacy issues." (G7)

"An incentive that goes from the pharmacy saving directly to the pocket of the physician is disreputable. If people knew of it..." (G5)

In some geographical areas, such as Catalonia, where GPs are *economically penalized *for non-compliance with budgetary constraints, the application of these measures has had very negative results. More specifically, the GPs describe the system as a de-motivating measure that can result in deviance in decision-making, thus distancing the physician from the needs of the patient. In addition, they criticize the fact that GPs are awarded according to short-term evaluations, ignoring those whose "savings" are sustained over the long term.

"I do my work well, that is what I do day to day. If the glycaemia is 8.3, bad luck. My salary does not have to depend on whether the patient eats or does not eat according to our suggestions, this is up to him" (G8)

"We must be alert since incentives can become discouraging" (G6)

In the region of Aragon, where prescribing incentives are based on greater access to training activities, this measure is believed to need realignment, since it is precisely those GPs who are poorly evaluated who have the greatest need for training. In addition, continuous training is an entitlement for all healthcare professionals and, as such, should not be considered an award.

"I believe it should be the other way round. The training days should be given to those who do not reach a certain expenditure level" (G3)

In general, all of the groups highlighted the need to evaluate the *quality of drug prescription and the health care *they provide rather than the profits they generate, and emphasised on the relationship between the latter and the implementation of health education activities as part of daily clinical practice.

"We are not rewarded for doing things well, but for spending less" (G5)

"We already have a good measure of the pharmacy expenditure [...]. What we now need is to be able to tell a colleague whether he has worked enough or not, whether he is able to solve the problems [...] satisfactorily for him and for the patient. This is how we would obtain the perfect circle" (G7)

"Why do they talk about pharmacy instead of treatment? [...] Why isn't the psychological treatment taken into account together with the prescription of anti-depressants?" (G5)

### Proposals for effective drug cost control

The proposals offered in the sessions focused on three axes involving a) the health administration, b) specialist care and c) the pharmacy setting.

a) Opinions regarding health administrators' responsibilities

The principal proposed measure was to eliminate from the list of medications funded by the public administration drugs whose prescription carries penalties under the incentive schemes (for example, "new medications"). The development of cooperative guidelines such as consensus vademecums, executed as a collaboration between PC and specialist care, is a possible route for implementing this control mechanism. GPs also suggested that the databases of their computer applications be adjusted to limit the number of drugs listed and to include information on the retail prices of the medications.

Another aspect of general concern is the need to *extend training *not only to PC teams but also to specialist physicians, pharmacologists and citizens accessing PC. GPs point out that there should be guarantees that these training activities are totally independent of the pharmaceutical industry, that they are taught by experts, and that they offer the opportunity to discuss physicians' prescription practices in a team atmosphere.

"I believe that the specialists need to be trained more [...]. In this way, we would probably observe a quantitative and qualitatively much better evaluation" (G1)

With respect to prescribing incentives linked to the rational use of drugs, the consensus was that these mechanisms should involve time within physician's daily medical practice assigned to professional development and organization. Incentives must not constitute awards for performing the job, but must instead be a *stimulus to continue doing their job well*. In this sense, they all agree that there should be an emphasis on promoting spaces for group discussions oriented towards the improvement of their clinical competences.

"And we need to be given internal incentives, we need to receive continuing training, we have to be more content, they have to resolve the deficiencies so that we can attend the patients, they have to repair our work-places, we have to be able to prepare our sessions here, not in our own houses...This is what actually encourages us" (G6)

In any event, most GPs suggest that incentives need to be personalized, taking into account the aspects that motivate each of them. To make this possible, some of them demand the *self-management *of the pharmaceutical budget by each HC itself.

"Let us be self-managed, we would then discuss what motivates us ourselves, and we would doubtless know how to distribute the awards" (G5)

"If health centres were private and we were told: here is your budget [...], pharmacy-related issues would doubtless be our priority. Why? Because this would enable us to take action over certain matters on which we cannot act right now, such as specialist-initiated prescribing, among others" (G5)

Other suggestions that received a high level of agreement were the need to *involve the nurse sector *in patients' health education so as to improve treatment adherence and the well-known requirement that the time-to-patient ratio of the consultations be increased.

"They are focusing on drug prescription issues with a massive power. In contrast, other aspects such as therapeutic aptitudes etc. are left aside, with absolutely no value placed on other actors that play a role here...We are not healthcare professionals working isolated, so how do they evaluate the weight of the nursing sector?" (G5)

b) Opinions regarding specialist physicians' responsibilities

All GPs identified their *specialist colleagues *as being an important part of drug cost control. The participants raised two main measures of special relevance in relation to the rational use of drugs: 1) considering specialist physicians as potential candidates for continuous training activities and 2) providing them with a better understanding of drug cost containment measures. Furthermore, as occurs in PC, their incentive schemes should focus on the level of fulfilment of the budget containment objectives, rather than on the interests of the pharmaceutical industry.

" [...] Everybody must be treated in exactly the same way, [...], working well should be measured equally everywhere" (G8)

c) Opinions regarding the responsibilities of the pharmacists

Part of the responsibility for drug cost control falls on the *pharmaceutical setting*. In view of the prescription modifications carried out by pharmacists, GPs consider it necessary that a "strong" policy of regulation be implemented. They point out that the pharmaceutical setting obtains an economic benefit as a consequence of prescription modification, for which the health system should be compensated.

"I believe pharmacists are responsible for always giving the patient one same drug" (G1)

" [...] As a result, the pharmaceutical setting makes millions of euros and this is not right. [...]. It is the second element to be blamed ..." (G8)

A novel suggestion captured in several interviews is that drug dispensaries be located in the HC, as is the case in other countries. All GPs strongly believe that this measure would ensure that only the necessary dose would be dispensed, avoiding the under-usage or wasting of excess drugs because of inappropriate packaging or changes in the prescription on the part of the pharmacist.

## Discussion

Our results demonstrate that, at present, GPs are conscious of their public responsibility with respect to pharmaceutical costs. This result suggests a shift from the findings highlighted by Denig P et al in the 90's [12], in which the relative cost of a prescribed drug did not, as yet, constitute a priority for the healthcare professionals working in PC. Moreover, the clash between health economics and clinical freedom observed among clinicians mentioned by the late health economist Alan Williams two decades ago [28] seems to fade away gradually. Nevertheless, these changes coexist with the deeply rooted GP's ideological focus on individual patient's needs and the quality of the given care [19,20,29].

Besides, the current control mechanisms give rise to feelings of impotence and injustice among GPs--mainly because of factors that are beyond their remit. Accordingly, the results of a study regarding a representative sample of Spanish GPs indicated that one out of every three considered their decisions to be conditioned by factors other than their knowledge and judgment [30]. These factors include their relationships with patients and the implication of their colleagues from specialist care.

Regarding patient-related factors, the pressure that they can occasionally exert in order to obtain medications that "cure" their health problem has been discussed previously [20,31]. This problem is aggravated when the amount of time available to attend to each patient is limited [32]. This pressure can be very difficult to handle because, in most cases, there is little time to explain the GPs preferred treatment option to dissenting patients [9].

As for the consequences of specialist-initiated prescriptions, previous research has shown that, after referral, the cost of medication rises by 23% [33] due to the use of more innovative and costly drugs [34]. Thus, specialist-initiated prescribing was strongly criticized by GPs in the study carried out by Prosser et al [20]. According to the latter, the effects of this include prescribing expensive formulations and branded drugs, early uptake of new, expensive drugs, recommending a named drug rather than a class of drug from which GPs could select a cost-effective alternative, pharmaceutical industry promotion, cost-shifting from secondary to primary care and price differentials between the hospital and community.

Another issue that leads to a lack of motivation among the GPs is related to the "what" and "how" of drug cost control. The interviewed physicians believe it necessary to put a higher value on activities with low marginal output, such as health education activities, due to their contributions to better prescription quality. González et al [11], in their Health Administration Manual, added that otherwise there is a risk that the GPs will spend their time on activities with higher marginal output, ignoring the objectives of their work organization. Our results also reveal the possible misuse and opportunist behaviour that financial incentives may inspire leading to conflicts of interest between the physician and the patient, among others [35]. Moreover, the economic penalties imposed on those who do not meet the budgetary targets set by the administration may lead to a potential deterioration of the work environment due to the tension that could arise among the PC teams [22].

Under these circumstances, the interviewees suggest several measures that, directly and indirectly, can contribute to a more efficient method of controlling the pharmaceutical budget. In the first place, they consider it essential that a greater degree of commitment be made both by specialist care and the pharmacy setting. Not only should there be a formal plan regulating their prescription habits through drug cost control mechanisms similar to those implemented in PC, but the "culture" of a more rational use of drugs should also be promoted in these settings, as it is also concluded by Prosser et al [20]. Carrying out collaborative meetings with specialist care or the development of joint drug lists or formularies are some of the actions taken by prescribing advisers in the UK to improve prescription management in PC [36].

Second, the GPs insist on the need to be in harmony with their work-mates through the development of spaces devoted to sharing insecurities and learning from the errors of others. This idea has previously been documented by Caamaño et al [37], who reported that teamwork in PC was associated with better prescription patterns. In this sense, the Primary Care Organisations (PCOs) established in England in 1999 could be a reference framework [38]. Money saved on prescribing costs is a collective benefit to all practices in the PCO and conversely, the penalties for over spending on drugs are only collective. In theory, GPs within a PCO collaborate for the greater good of all, acquiring a corporate ethos to accomplish health care reform [39].

The importance of the role played by the coordinator of the HC was also reported in the present study. The coordinator should represent the nexus of healthcare professionals and the administration, with the goal of facilitating participation of the former in the decision-making of the latter [15]. The willingness of PC physicians to participate in cost containment decisions, rather than be guided by administrative rules, was brought to light in a study carried out in four different European health care systems [40]. In UK, the role of PCO prescribing leads and advisers in developing cost-based prescribing strategy and incentive schemes is increasingly proactive [41].

With respect to prescribing incentive schemes, the interviewees assert the need for personalized measures adapted to each HC and physician that take into account their individual expectations as well as the influence of the measures on the quality and cost of their prescriptions [30]. Some of the GPs report that monetary incentives are not sufficient *per se*, and that self-management of the incentives should be the final goal. Nevertheless, a recent Cochrane review concludes that overall evidence for the effect of financial incentives, such as drug budgets, is weak: while drug spending and the volume of drugs prescribed decreases, there is not clear evidence about their effects on health care utilization (such as referrals to specialists) [13]. GPs also express the need to be encouraged by professional incentives (such as time available for training and research activities and, again, autonomy in budget management) and work incentives (technical resources leading to a better working environment in the HC). A previous report concurs with the concept that a balanced combination of economic and non-economic incentives, according to the interests of healthcare professionals, is more appropriate than the isolated use of financial incentives [11].

In relation to the rationalization of drug costs through cutbacks in the breadth of medications that are offered, the interviewees suggest several possible technical measures. They suggest exclusion of drugs whose prescriptions carry penalties according to the incentive scheme. The use of close-formularies containing a specific list of best-value drugs was also supported by physicians working in Veterans Affairs health care facilities, although access to nonformulary drugs and timely approval of requests of nonformulary medications were strong predictors of clinical satisfaction and support for cost containment measures [42]. Moreover, close-formularies run the risk of generating frustration and rejection by the public [43,44] and outcomes associated to its implementation are not always positive [45]. Consequently, this type of initiatives should be preceded by campaigns to increase awareness regarding the rational use of drugs and provision of information that often runs counter to patients' perceptions of the prescriptions.

Moreover, they support the inclusion of the retail prices for the drugs in the prescription database of the computer application. This demand responds to GP's lack of knowledge of actual drug prices, ascertained by Prosser et al [20]. They also assert that GPs rarely monitor costs or view prices when prescribing, despite having access to prices via computerized decision-support systems or paper sources [20].

Finally, the discussion with GPs focused on the importance of continuous training. The evidence suggests that educational outreach is among the most effective means of bringing about change [19,37,46], as long as the training schemes are derived from sources independent of the pharmaceutical industry [3,9].

As far as the potential limitations of the study are concerned, the most important one is the study sample, since GPs were selected on the basis of a quantitative study mentioned earlier. The use of a theoretical sample would have allowed for analysis of the influence of variables related to healthcare professionals or the organizational structure of the HC. As a result, we consider the present study as exploratory and its objective as being mainly to document the presented research questions in the most complete manner possible.

As far as a generalization of the research ideas is concerned, we believe that the number and the diversity of individuals in the final sample permit extrapolation of the results to subjects and contexts that are similar to those in the present study.

The frequency with which a consensus was reached among the focus groups supports the assumption that saturation of the information was reached. Nevertheless, the common elements of the GPs' training, their tendency to respond according to the phenomenon of social acceptability, their awareness that some expectations can sound disproportionate, and their link with the PC setting may limit, only to a certain degree, the sincerity of the responses and the representiveness of the results.

## Conclusion

Principal conclusions of the study were defined by the participating GPs. They are conscious of their public responsibility with respect to pharmaceutical costs, but highlight the need to spread responsibility for cost control among the different actors of the health system. They insist on implementing measures to improve the quality of prescriptions, avoiding mere quantitative evaluations of prescription costs. They also suggest moving towards the self-management of the pharmaceutical budget by each HC itself, as a means to design personalized incentives to improve their outcomes. Finally, it was concluded that this plan is the only way to pre-empt the feelings of injustice, impotence, frustration and lack of motivation that currently exist among GPs as a result of the implemented measures.

These proposals need to be considered by the health administration as one method of achieving a more rational use of drugs. Future research should focus on aspects such as the cognitive mechanisms underlying physician's behaviour, as studied by Godin et al [47], in order to incorporate cost considerations into GP decision making. The analysis and comparison of the effectiveness of methods that facilitate the convergence of objectives and priorities between clinicians and administrators, in terms of drug cost control mechanisms, is another issue worth studying [48]. Results of this research work may throw light on how prescribing support is best delivered and how implementation strategies are best combined.

## Competing interests

The authors declare that they have no competing interests.

## Authors' contributions

APT and ASM generated the research question. SML and BOB conducted the discussions with the focus groups, provided expertise on methodology and participated in the coding and analysis. ACL carried out the interpretation of the results and developed the discussion. APT, ACL and BOB contributed to the drafting of the manuscript. All authors have read and approved the final version of the manuscript.

## Pre-publication history

The pre-publication history for this paper can be accessed here:

http://www.biomedcentral.com/1472-6963/9/209/prepub
